# A Microfluidic Mixer of High Throughput Fabricated in Glass Using Femtosecond Laser Micromachining Combined with Glass Bonding

**DOI:** 10.3390/mi11020213

**Published:** 2020-02-19

**Authors:** Jia Qi, Wenbo Li, Wei Chu, Jianping Yu, Miao Wu, Youting Liang, Difeng Yin, Peng Wang, Zhenhua Wang, Min Wang, Ya Cheng

**Affiliations:** 1 State Key Laboratory of High Field Laser Physics, Shanghai Institute of Optics and Fine Mechanics, Chinese Academy of Sciences, Shanghai 201800, China; qijia@siom.ac.cn (J.Q.); liwb@shanghaitech.edu.cn (W.L.); 18722372031@163.com (J.Y.); yindf@siom.ac.cn (D.Y.); wangpeng2015@siom.ac.cn (P.W.); 2 University of Chinese Academy of Sciences, Beijing 100049, China; 3 School of Physical Science and Technology, ShanghaiTech University, Shanghai 200031, China; 4 XXL-The Extreme Optoelectromechanics Laboratory, School of Physics and Electronic Science, East China Normal University, Shanghai 200241, China; wumiao1993@126.com (M.W.); 15253172638@163.com (Y.L.); zhwang@phy.ecnu.edu.cn (Z.W.); mwang@phy.ecnu.edu.cn (M.W.); 5 School of Physics Science and Engineering, Tongji University, Shanghai 200092, China; 6 State Key Laboratory of Precision Spectroscopy, East China Normal University, Shanghai 200062, China; 7 Collaborative Innovation Center of Light Manipulations and Applications, Shandong Normal University, Jinan 250358, China

**Keywords:** ultrafast laser microfabrication, microfluidic, glass bonding

## Abstract

We demonstrate a microfluidic mixer of high mixing efficiency in fused silica substrate using femtosecond laser-induced wet etching and hydroxide-catalysis bonding method. The micromixer has a three-dimensional geometry, enabling efficient mixing based on Baker’s transformation principle. The cross-sectional area of the fabricated micromixer was 0.5 × 0.5 mm^2^, enabling significantly promotion of the throughput of the micromixer. The performance of the fabricated micromixers was evaluated by mixing up blue and yellow ink solutions with a flow rate as high as 6 mL/min.

## 1. Introduction

Mixing is one of the dominating processes in chemical reactions and analyses. With microfluidic technology, various schemes have been conceived and implemented to realize highly efficient mixing of liquids by manipulating micro- and nanoscale fluids in sophisticated manners [1,2,3,4,5,6]. The geometries that have been incorporated into the microfluidic channels for promoting mixing efficiency include T-shaped microchannel, H-shaped micromixer, and grooved micromixer, etc. [7,8,9,10,11,12]. In particular, it has been demonstrated that a three-dimensional (3D) passive micromixer, which was designed basing on the Baker’s transformation concept, can enable fast and efficient mixing even in the low-Reynolds-number condition [13]. Many planar manufacturing approaches, such as casting or injection molding, have been adopted to fabricate on-chip micromixer devices. However, these methods are inadequate for three dimension (3D) complex structures fabrication. 3D printing technology, which can be employed to produce 3D structures, always uses organic materials (i.e., epoxy resin). The organic materials are unsuitable for some microfluidic devices fabrication, since they are easily modified or dissolved by chemical reagents and damaged in high-temperature or high-pressure environments. In contrast, glass materials are chemically stable and resistant to corrosion, high temperature and pressure, making them excellent candidates for microfluidic chips preparation and functionalization. As a maskless technology, femtosecond laser direct writing (FLDW) enables rapid prototyping and provides a straightforward approach to fabricate 3D structures inside photosensitive materials, including polymer and glass. The capability of 3D prototyping with high resolution in a wild range of transparent materials makes FLDW a promising and superior technology for the fabrication of microfluidic devices. It should be mentioned that the 3D micromixer was fabricated using FLDW of glass [14,15,16,17,18], which has been proved to be a straightforward approach of fabricating 3D microfluidic structures and integrated optofluidic devices [19,20,21,22].

The femtosecond laser micromachining can be an ideal tool for fabricating such structures owing to its high fabrication precision and 3D capacity. However, the micrometer scale mixers suffer from a relatively low throughput for various kinds of chemical reactions. The solution is to fabricate microfluidic mixers consisting of relatively thicker and longer channels, which is nevertheless challenging for the current state-of-the-art femtosecond laser-induced selective etching (FLSIE) technique [23].

Here, we demonstrate the fabrication of 3D micromixers of large footprint sizes in glass using femtosecond laser micromachining. We improve the fabrication efficiency by optimizing the laser pulse duration. The 3D micromixer, designed basing on the Baker’s transformation, is constructed by bonding two substrates with complementary microfluidic channels fabricated on the surface. The advantage of our design is that for the upper and lower halves of the mixer, they both have a 2D planar geometry, which facilitates obtaining the designed structures by FLSIE technique, while the mixer produced after bonding has a true 3D geometry to implant the Baker’s transformation operation. The elimination of any vertical structures in the upper and lower halves of the micromixer provides more tolerance for the alignment between the upper and lower halves during the bonding; while the employment of Baker’s transformation for the mixing leads to high mixing efficiency regardless of the flow rates in the microchannels. Compared to the previous micromixers designed and achieved basing on Baker’s transformation, the throughput of our micromixer can be significantly enhanced. As shown in Table 1, we compared the performances of different micromixer devices in detail.

## 2. Device Design and Numerical Simulations of Mixing Process

**Figure 1:**
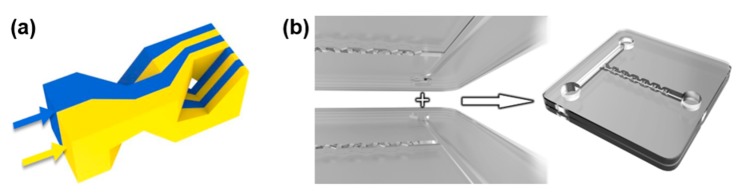
(**a**) Schematic view of working mechanism of the micromixer. The two microfluidic streams sent into the mixing unit are divided into four sub-streams alternatively spaced with each other at the middle of the unit and further divided into eight streams at the exit; (**b**) Schematic of the 3D micromixer constructed by bonding two substrates with microfluidic channels fabricated by femtosecond laser micromachining on the top surfaces.

Figure 1a illustrates the mixing effect in a single mixing unit. The unit can be arranged into a chain for the construction of a high efficiency and high throughput microfluidic mixer. The mixing unit features a 3D microstructure which splits, routes, and reorganizes the fluidic streams within the upper and lower halves of the channel into an array of alternatively arranged sub-streams, which is the so-called Baker’s transformation. In such a manner, the number of microfluidic streams can be quadrupled after they pass each of the mixing unit. The working mechanism provides an efficient way of mixing with the relatively simple geometry as compared to that in [13,14]. The design of the whole micromixer is schematically illustrated in Figure 1b. First, the upper half of the micromixer is engraved into the fused silica substrate using femtosecond laser-assisted chemical wet etching. Likewise, the lower half of the micromixer is fabricated using the same technique. The two substrates are finally bonded into the micromixer using a hydroxide-catalysis bonding technique as described later in this paper.

**Figure 2:**
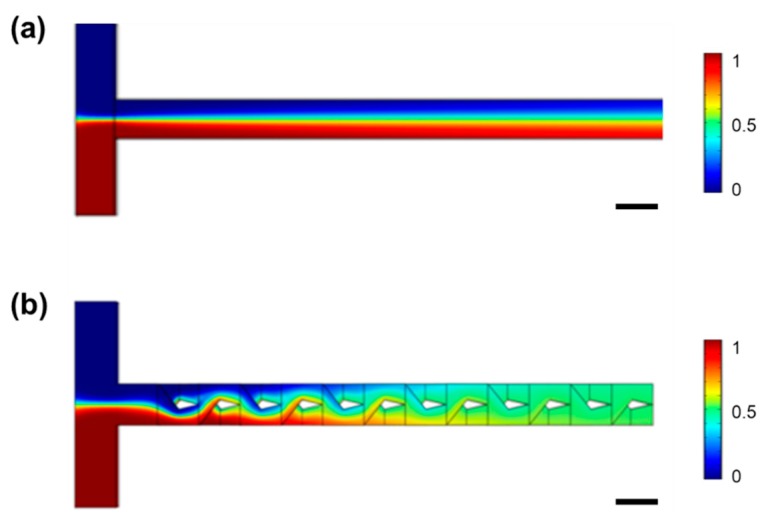
Numerical simulations of mixing performances at a flow rate of 2 mL/min in (**a**) a T-shape straight microchannel with a rectangular cross section and (**b**) a 3D micromixer consisting of six mixing units. The straight microchannel and the 3D micromixer have the same total length and the same cross section area. Scale bar in (**a**,**b**): 0.5 mm.

The mixing performance of the designed 3D micromixer composed of six mixing units is numerically simulated by solving the microfluidic incompressible Navier-Stokes and convection diffusion equations using a finite element analysis software (COMSOL Multiphysics 5.4, COMSOL Multiphysics GmbH, Göttingen, Germany). The simulations results compared with that of a straight microfluidic channel are illustrated in Figure 2. The two structures are of the same cross-sectional size and total length. One can see that in the 1D straight channel in Figure 2a, mixing only occurs at the interface of two streams as a result of diffusion. Owing to the laminar flow, which dominates at low Renolde numbers in the microfluidic channels, the overall mixing efficiency is low. In contrast, the 3D mixer in Figure 2b shows an excellent mixing effect thanks to the working mechanism described above.

## 3. Fabrication of the 3D Micromixers

The 3D micromixer is fabricated using femtosecond laser-assisted chemical wet etching technique and the hydroxide-catalysis bonding method. First, the upper and the lower half of the micromixer is engraved into the fused silica substrate using femtosecond laser-assisted chemical wet etching. The femtosecond laser pulses (1030 nm, up to 400 µJ, 270 fs) were provided by a commercial femtosecond laser source (Pharos, Light Conversion Ltd., Vilnius, Lithuania). The duration of the laser pulse can be tuned from 270 fs to 15 ps by adjusting the distance between the gratings in compressor. After passing through an attenuator and a beam expanding system, the laser pulses were then focused into the fused silica glass using an objective lens (Olympus MPLFLN, 10×, NA = 0.3, OLYMPUS, Tokyo, Japan). A motion stage (ANT130-110-L-ZS, Aerotech Inc., Pittsburgh, PA, USA) was used to translate the objective lens along Z direction to control the depth of the focus, and the fused silica glass sample was mounted on an XY motion stage (ABL15020WB and ABL15020, Aerotech Inc., Pittsburgh, PA, USA) and smoothly translated with a positioning precision of 100 nm. Both the translation stages were controlled using a high-performance motion controller (A3200, Aerotech Inc., Pittsburgh, PA, USA). In our fabrication, the repetition rate of the laser was set to 100 kHz, and the laser pulse duration was set as 4 ps [25,26]. The laser focal spot was scanned along the pre-designed paths layer by layer with a layer spacing of 10 µm to produce the microchannels on both glass substrates. The scan process was performed from the bottom to the top of the substrate, and the scan speed was fixed at 10 mm/s.

After laser irradiation, the glass samples were immersed in a solution of potassium hydroxide (KOH) with a concentration of 10 mol/L to selectively remove the glass material irradiated by the laser pulses. The microchannels on both glass substrates can form after the etching in KOH solution.

**Figure 3:**
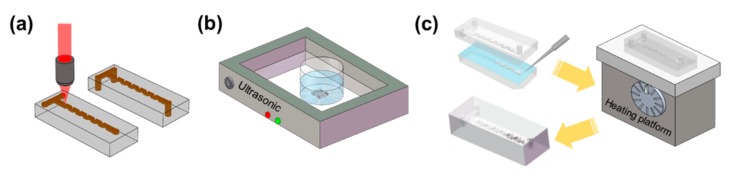
Fabrication procedures: (**a**) ultrafast laser direct writing; (**b**) chemical wet etching; and (**c**) hydroxide-catalysis bonding.

Lastly, the two substrates were combined into the micromixer using the hydroxide-catalysis bonding method. First, the top surfaces of the two glass substrates were polished, and the two substrates were ultrasonically cleaned in acetone for 10 min and subsequently in distilled water for 10 min. Then, a drop of 2% sodium hydroxide (2% NaOH) solution was applied to the bonding surfaces of the two glass substrates. Afterwards, we carefully adjusted the position of the two glass substrates under the transmission illumination microscope to ensure an accurate alignment between the microchannels engraved in the two substrates. After that, the whole sample was squeezed gently, held for 24 h at the room temperature, and then annealed at 200 °C for another 24 h to reinforce the bonding strength [27,28]. The procedures of the micromixer fabrication are illustrated in Figure 3.

## 4. Results and Discussion

**Figure 4:**
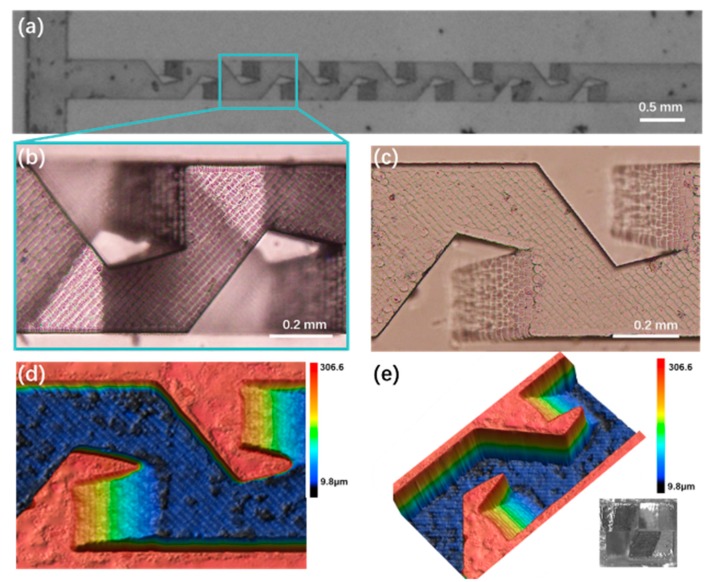
Top view optical micrograph of (**a**) fabricated micromixers; (**b**) the detailed features of mixing unit; (**c**) the microchannel on half glass substrate before bonding. (**d**,**e**) are the 3D images of (**c**) from different view angles captured by laser confocal microscopy. Inset: Image of the cross section at the end of the mixing unit after bonding.

The top view micrograph of the fabricated 3D microfluidic mixer is shown in Figure 4a. One can see that it contains six mixing units. The sharpness of the edges and corners in the fabricated structure provide the evidence of the high machining quality of the femtosecond laser. Figure 4b presents the detailed top view image of one of the mixing units. Figure 4c presents the top view image of half of the micromixer before bonding. Figure 4d,e exhibit the 3D profiles of the structure in Figure 4c from different angles of view, showing a maximum depth of ~270 µm in the fabricated microchannel. The optical micrograph of the cross section was illustrated in the inset of Figure 4e. The cross section shows a square profile with a side length of 0.5 mm. Because of the relatively large height and width of the micromixer, the production throughput can be efficiently promoted, which is highly in demand by industrial application. It should be noted that the measured roughness of the micromixer’s inner surface in the region of 200 µm × 200 µm is 872 nm, which is orders of magnitude smaller when compared to the size of the micromixer. As a consequence, the impact of the surface roughness to the mixing process can be ignored.

**Figure 5:**
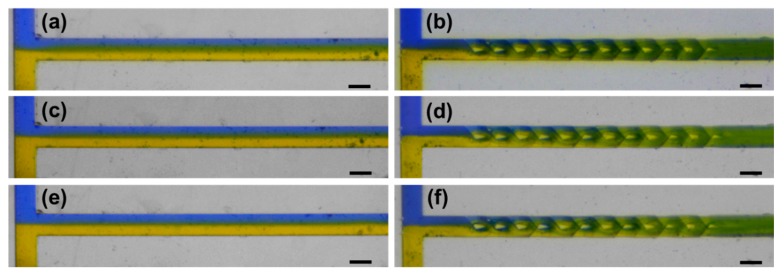
Microscope images of the mixing behaviors of the blue and yellow ink solutions in straight channel at a flow rate of (**a**) 1 mL/min, (**c**) 2 mL/min and (**e**) 6 mL/min, and in fabricated 3D micromixer at a flow rate of (**b**) 1 mL/min, (**d**) 2 mL/min and (**f**) 6 mL/min. Scale bar in (**a**–**f**): 0.5 mm.

At last, we experimentally demonstrated the mixing of the fabricated 3D micromixer using two kinds of ink of blue and yellow colors, as shown in Figure 5. The experimental performances of the 3D micromixer compared with that of the straight microchannels were conducted at three different flow rates of 1 mL/min (Figure 5a,b), 2 mL/min (Figure 5c,d), and 6 mL/min (Figure 5e,f), which correspond to Reynolds numbers of 8.33, 16.67, and 50 in the micromixer respectively. It can be seen that in the straight channel, the mixing efficiency contributed by diffusion decreases to the increasing flow rate. Overall, the mixing effect in Figure 5a–c are much weaker than that in the 3D mixer as shown in Figure 5b,d,f respectively. Interestingly, as the mixing in the 3D micromixer is achieved dominatingly by the Baker’s transformation mechanism but not by diffusion, the mixing performances at different flow rates appear similar to each other. The stabilization of mixing efficiency at the variable flow rate provides a controllable way to manipulate chemical/biological reactions in the microchannel by only changing the flow rate, which leads to a predictable change of the dwell time of the reactants in the microchannel.

## 5. Conclusions

To conclude, we have designed and fabricated a 3D microfluidic mixer based on femtosecond laser micromachining technology and hydroxide-catalysis bonding method. Both our simulation and experimental results show that the device can realize efficient microfluidic mixing. The compact and efficient 3D micromixer can be used in applications ranging from chemical/biological analysis and microfluidic synthesis of materials to fine chemistry microreaction. Our technique also has promising potential in electrophoretic and some other relative applications [29] because it can offer the capabilities of 3D fabrication, controllable fluidic throughput and multifunctional integration.

## Figures and Tables

**Table 1:** Comparison of three different types of micromixer devices designed and fabricated basing on the Baker’s transformation.

Channel Width × Height	Maximum Flow Rate	Materials	Processing Method	Ref.
160 µm × 20 µm	~200 µL/min	PDMS	PDMS molds	[24]
50 µm × 75 µm	~20 µL/min	Porous glass	Femtosecond laser direct writing and annealing	[14]
500 µm × 500 µm	6 mL/min	Fused silica	FLISE and bonding	This work
